# Infective Endocarditis Manifesting as Severe Elevation in Serum Aminotransferases in the Absence of Severe Tricuspid Regurgitation, Heart Failure, or Shock: A Diagnostic Challenge

**DOI:** 10.7759/cureus.16044

**Published:** 2021-06-29

**Authors:** Eluwana A Amaratunga, Jason A Hoggard, James Kamau, Emily B Ernst, Mathai Chalunkal, Richard Snyder

**Affiliations:** 1 Internal Medicine, St. Luke’s University Health Network, Easton, USA; 2 Congenital Heart Surgery, Texas Children's Hospital, Houston, USA; 3 Internal Medicine, St. Luke's University Health Network, Easton, USA

**Keywords:** transaminitis, elevated liver associated enzymes, liver injury, infective endocarditis, blood culture negative endocarditis, blood cultures, duke criteria, aminotransferases

## Abstract

Infective endocarditis (IE) is a challenging condition to diagnose, given its protean clinical signs and symptoms, Elevation in serum aminotransferases in IE is associated with valvular regurgitation, acute heart failure, or congestive hepatopathy. Studies show co-existing liver failure portends worsening outcomes in IE and poses a challenge for successful surgical management. Here we report a diagnostic challenge in a 35-year-old man with IE presenting predominantly with gastrointestinal symptoms and severe elevation in serum aminotransferase. The degree of aminotransferase elevation in our patient prompted consideration of alternative causes like acetaminophen toxicity. Severe elevation in aminotransferases as an initial presentation in the absence of significant valvular regurgitation, acute right heart failure, or shock is uncommon. A high degree of suspicion is required to diagnose IE when patients present with atypical signs and symptoms to avoid delay in initiation of antibiotics and improve overall morbidity and mortality.

## Introduction

Infective endocarditis (IE) is a relatively common condition with an incidence of 3 to 10 per 100,000 persons per year and rising [1-3]. Due to its nonspecific symptoms and signs on presentation, this diagnosis is often delayed resulting in increased morbidity and mortality. The classic clinical presentation of fever of unknown origin only represents a minority of diagnosed cases while other nonspecific symptoms such as malaise, loss of appetite, dyspnea, cough, weight loss, and embolic phenomena tend to predominate either in isolation or in combination [4,5]. Associated liver enzyme elevation is usually identified when IE is complicated by tricuspid and/or mitral regurgitation and acute right-sided heart failure, severe congestive heart failure causing congestive hepatopathy, or septic shock with a component of ischemic hepatitis [6-8]. The presence of liver failure in IE has been demonstrated to portend worsening outcomes, especially for valvular surgery [6,9]. We report a case of IE with an initial presentation of predominantly gastrointestinal symptoms with severe elevation in aminotransferases without evidence of right-sided heart failure, congestive hepatopathy, or septic shock. Despite having a history of hepatitis C and intravenous drug use (IVDU) in this patient, the initial presentation was extremely atypical such that the initial management focus was for possible acetaminophen toxicity. We highlight the diagnostic challenge(s) posed by this patient which led to a delay both in diagnosis and in antibiotic initiation.

## Case presentation

A 35-year-old man with a history of IVDU and chronic hepatitis C presented with epigastric pain, nausea, and diarrhea for four days. He denied fever, chills, chest pain, dyspnea, orthopnea, or paroxysmal nocturnal dyspnea. He took four tablets of acetaminophen 325 mg for abdominal pain one day before presentation with no relief. On admission, he met the criteria for systemic inflammatory response syndrome (SIRS) with tachycardia (113 beats per minute), tachypnea (20 breaths per minute), leukocytosis of 19.89 thousand/uL white blood cells, and a lactic acidosis of 3.8 mmol/L. He was afebrile. Blood pressure on admission was 120/56 mmHg and remained stable. The examination was significant for generalized abdominal tenderness without guarding or free fluid. There were no audible heart murmurs or S3 on auscultation. His lungs were clear to auscultation with reduced breath sounds in the right lower lung field. No jugular vein distention (JVD) was noted. He had warm peripheries and no lower extremity edema. There were no skin lesions or rash noted. Laboratory studies revealed elevated serum aminotransferases with aspartate aminotransferase (AST) 733 U/L, alanine aminotransferase (ALT) 1286 U/L, international normalized ratio (INR) of 1.59, and total bilirubin of 0.79 mg/dL. Platelet count was 162 x 10^3^/uL and hemoglobin was 10.5 g/dL. Creatinine was 1.52 mg/dL, elevated from his baseline of 0.8 mg/dL. Serum acetaminophen level was within normal limits at 4.5 ug/mL, alcohol level was undetectable, and urine toxicology was positive for methamphetamines and opiates. A contrast-enhanced CT scan of the chest/abdomen/pelvis showed hepatosplenomegaly without evidence of hepatic congestion. The right upper quadrant ultrasound showed hepatomegaly with no evidence of portal or hepatic vein thrombosis.

**Figure 1:**
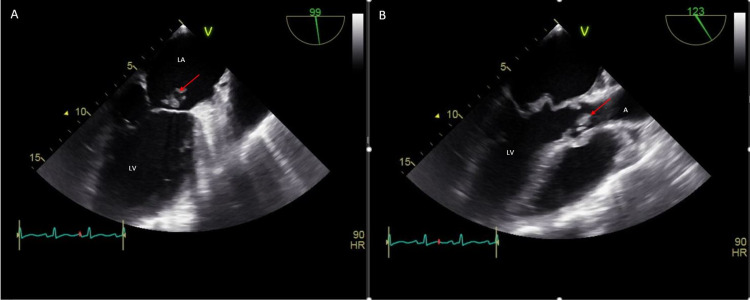
Transesophageal echocardiogram (TEE) images demonstrating a large mitral valve vegetation (A) and a medium-sized aortic valve vegetation (B) LA = left atrium, LV = left ventricle, A = aorta

**Figure 2:**
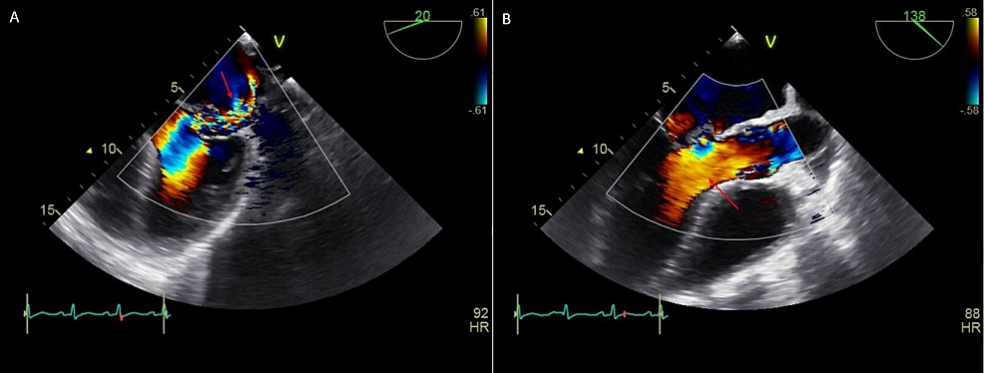
Color Doppler Transesophageal echocardiogram (TEE) demonstrating moderate-severe mitral regurgitation (A) and moderate aortic regurgitation (B)

As no obvious source of infection was identified, he was monitored off antibiotics. Intravenous (IV) N-acetylcysteine was initiated with IV normal saline due to concerns of delayed acetaminophen toxicity despite normal acetaminophen levels. Workup for acute liver injury including acute viral panel and autoimmune hepatitis panel were unremarkable except for a positive hepatitis C antibody. Despite treatment, AST and ALT levels continued to increase to 2719 U/L and 3003 U/L, respectively. The etiology of worsening aminotransferase elevation was unclear at this point. The possibility of methamphetamine toxicity was considered. On day two of admission, blood cultures grew gram-positive cocci bacteria, which were later identified as *Enterococcus faecalis*. Broad-spectrum IV antibiotics were initiated. Transesophageal echocardiogram (TEE) showed a large vegetation on the mitral valve with perforation and medium-sized vegetation on the aortic valve (Figures 1A-1B). There was moderate-severe mitral regurgitation and moderate aortic regurgitation (Figures 2A-2B). Left and right ventricular functions were preserved. Due to the presence of ongoing liver failure and extensive valvulopathy requiring major cardiac surgery, he was transferred to a tertiary facility for further management by cardiothoracic surgery.

## Discussion

IE is defined as an infection in the endocardium or heart valves. The modified Duke criteria for IE remains an important tool in the diagnosis, although the sensitivity of this is relatively low (Table 1). A prospective observational study done by Topan et al., to evaluate the individual value of each criterion of this tool, showed that the overall sensitivity is 63.23%. They further highlighted that the major criteria had a greater impact on the diagnosis, with echocardiography providing the most substantial contribution in confirming the diagnosis [10]. A high index of suspicion, however, is required to prompt a provider to perform an echocardiogram as this is not a routinely performed study. This is especially important in blood culture-negative infective endocarditis (BCNE). Blood cultures require a minimum of 24 hours of incubation and the presence of BCNE is not uncommon, making an early diagnosis challenging. The diagnosis is especially difficult when patients present with atypical signs and symptoms. The classical presentations of pyrexia of unknown origin, classical Osler nodes, or Janeway lesions are scarce, as in the case of our patient [5,10].

**Table 1:** The modified Duke criteria HACEK = *Haemophilus* spp.,* Actinobacillus actinomycetemcomitans, Cardiobacterium hominis, Eikenella* spp., *Kingella kingae*; IE = infective endocarditis, IVDU = intravenous drug use, IgG = immunoglobulin G Diagnosis of IE includes two major criteria, or one major criterion + three minor criteria, or five minor criteria [10].

Major criteria	Minor criteria
Positive blood cultures for IE:	Predisposing heart condition or IVDU
Positive for typical microorganisms consistent with IE (Viridans streptococci, *Streptococcus bovis*, HACEK group, *Staphylococcus aureus*, or community acquired enterococci) from two separate blood cultures, or two blood samples drawn 12 hours apart, or three, or a majority of at least four separate cultures of blood with first and last samples drawn 1 hour apart, or	Temperature >100.4F
Single blood culture positive for *Coxiella burnetii* or antiphase IgG antibody titer >1:800.	Vascular phenomena:
Endocardial involvement:	Major arterial emboli, septic pulmonary infarcts, mycotic aneurysm, intracranial bleed, conjunctival hemorrhages, and Janeway lesions.
Oscillating intracardiac mass on valve or supporting structures, in the path of regurgitant jets, or on implanted material in the absence of an alternative anatomic explanation, or	Immunologic phenomena:
Abscess, or	Glomerulonephritis, Osler nodes, Roth spots, and rheumatoid factor.
New partial dehiscence of prosthetic valve, or	Microbiologic evidence:
New valvular regurgitation.	Positive blood culture that does not meet the major criteria or serologic evidence of active infection with organism consistent with infective endocarditis.

Our patient presented with predominantly gastrointestinal symptoms of epigastric pain, nausea, and diarrhea and was found to have worsening aminotransferase elevation. The presence of elevated serum aminotransferases, with recent acetaminophen use, prompted the providers to initially treat him with a diagnosis of presumed acetaminophen toxicity despite a normal acetaminophen level. Other diagnostic considerations included elevation in serum aminotransferases secondary to severe sepsis superimposed on chronic hepatitis C, the source of the sepsis initially unclear. The degree of elevation in aminotransferases in the absence of hypotension prompted consideration of alternative causes such as toxin-induced, acute viral, and autoimmune hepatitis over IE. Furthermore, the absence of fever and other classic signs of IE and lack of acute heart failure made the suspicion of IE less likely. An echocardiogram was not performed initially as the patient had no signs of cardiac involvement and this delayed the initiation of antibiotics. With blood cultures becoming positive 24 hours later, an infectious etiology became more apparent.

Severe elevation in aminotransferases as an initial presentation of IE in the absence of signs and symptoms of right-sided heart failure is uncommon. Aminotransferase elevation is a nonspecific sign and has multiple etiologies of which viral and toxin-induced are the most common. Acetaminophen, halothane, isoniazid/rifampicin, sodium valproate, and sulfonamides are some of the medications that are known to cause elevated liver enzymes [11]. Viral etiologies include acute and chronic hepatitis, human immunodeficiency virus (HIV), Epstein-Barr virus (EBV), cytomegalovirus (CMV), human herpesvirus (HHV), and dengue fever. Other infectious causes include syphilis, leptospirosis, Lyme disease, rocky mountain spotted fever, fungal infections, and parasitic infections. Autoimmune causes (systemic lupus erythematosus) and systemic disorders (Wilsons’s disease, hemochromatosis, amyloidosis) are rare, but well-known causes of elevated aminotransferases [7,8]. In sepsis, the elevation of liver enzymes is only modest, however, if this coexists with septic shock, it could result in a striking elevation due to hypoxic liver injury as the liver is highly sensitive to reduction in blood flow [7,8]. Aminotransferase elevation in IE is commonly related to congestive hepatopathy [6]. Valvular injury in IE can lead to acute heart failure, pulmonary hypertension, and hepatic congestion. In the absence of shock, this would cause only mild-moderate elevation of aminotransferases [7]. As signs and symptoms of acute right heart failure are non-specific, it is important to perform an echocardiogram if the etiology of elevated aminotransferases is unclear, as hypoxia/ischemia and hepatic congestion can be easily overlooked [11].

While the prognosis of IE is dependent on multiple factors including patient age, type of organism, degree of valvular damage, and presence of complications like embolism or acute heart failure, studies have further shown that the duration of endocarditis can significantly affect the overall outcome [5]. Thus, timely diagnosis and management are important for better prognostic outcomes. Despite optimal medical management and advancement of surgical techniques, overall mortality for IE is generally high approaching close to 30% at one year, with no improvement over the past years [1,12], possibly due to the challenges in diagnosing the condition early in the disease process.

## Conclusions

The diagnosis of IE remains challenging especially when the clinical presentation is atypical often leading to delayed initiation of antibiotics. The presence of severe elevation in serum aminotransferases without evidence of shock or acute heart failure is an uncommon presentation of IE, and clinicians should be cognizant during such clinical presentations. An echocardiogram is needed for patients in whom there is a high clinical suspicion to confirm the diagnosis and prevent further delay of treatment, especially when the etiology of aminotransferase elevation is uncertain. The challenge of diagnosing IE early enough is likely contributing to the lack of improvement of overall mortality in IE despite advances in medical treatment and surgical intervention(s).
